# Fevers

**Published:** 1872-07

**Authors:** T. A. Cooke

**Affiliations:** Louisiana


### FEVERS.

BY T. A. COOKE, M. D., OF LOUISIANA.

We are in the habit of dignifying the phenomena, the events, the manifestations of life, of disease, and the impressions of agents upon the human body, as facts. We recoil from the truth that a fact in medicine, without an exception or condition, can hardly be said to exist. In the exact sciences our information is positive; but in medicine conditions or exceptions attach to all observations. Ipecacuanha will not always cause vomiting, nor opium sleep; out of all therapeutic facts, the most reliable are the emetic properties of the one, the soperific of the other. So with regard to the nature of inflammation: a morbid process, to which from time immemorial, most of the ill of flesh, are (very loosely) attributed, there exist in this day, as much disputation and ignorance as in any preceding age. Instead of being the cause, it is most probably always in its origin a symptom of disease. Who will say that in erysipelas the inflammation is the cause, and not the result of the general disturbance? a pure sequela, which sometimes becomes a disease productive of most serious evils?

No local inflammation can ever take place before the normal relations between the solids and fluids of the part are affected—perverting nutritive actions, accelerating, retarding, or suspending elimination in the part affected; nor can we have essential fever without an evident perversion or modification of these same movements—as is evident in typhoid and bilious, and, especially, in yellow fever. The grade or character of the inflammation depends upon the predominance of some one of the conditions mentioned, resulting from particular causes—a peculiar diathesis or temperament, or the presence of elements in the body which give a peculiar character to the secretions.

So that we have various kinds of inflammation, presenting untold degrees of benignity and malignity—an affection always, in my judgment, symptomatic, but which often in its turn becomes a disease, reacting upon the system with dangerous and sometimes fatal severity.

A statement, recently met with, that the redness of the blood seen in blood vessels leaving seats of inflammation, shows that there has been going on a process of suboxidation of this fluid in the part inflamed, has led me to much reflection. Whether correct or not, it is a statement full of interest to the physiologist as well as the pathologist. It appears very clearly to me, that this arterial color of the blood is not due to a process of suboxidation, but to the absence of deoxidation of the blood in the inflamed parts—that in inflammation neither atrophy or hypertrophy necessarily results. There may be swelling from effusion, pain from pressure, and the involvement in excited action of the nerves of the part, and redness—and this in its different grades will correspond with the molecular or excretery actions going on in the part—varying from a dusky redness to a bright arterial hue. The general phenomenon is that of redness—the blood retaining (neither more nor less) its arterial hue. So long as the arterial blood penetrates and traverses the inflamed tissue, redness will, under ordinary circumstances, predominate, because there are no effete matters eliminated—because carbonaceous matters, or those elements or compounds which unite with oxygen are locked up, as it were, in the inner chambers or cells of the parts, thus leaving the arterial blood unchanged at the time of leaving the inflamed tissue.

Such appears to be a correct explanation of the color of redness in inflammation—of the color of the blood both in the seat of the inflammation and in the vessels departing therefrom. However particular influences may modify the disease considered, its general phenomena are all reconcileable with the theory above proposed.

The death of the part from strangulation—from the effusion of corroding secretions, the breaking down of tissue consequent upon the free discharge of deleterious fluids, the production of pus, its absorption, &c., are all ultimate effects of some form of inflammation, but they impinge in no way upon the proposition that redness in inflammation, and of the blood of the part, is due entirely to the diminution or suspension of elimination in the part diseased.

It must at once strike the reflecting mind, that if this proposition embrace a truth—and we know of nothing in our art that more nearly approximates the dignity of a fact—it is one of great and paramount importance in practical medicine. We see in the invasion of all ardent fevers—in bilious fevers—especially in yellow fever—that venous blood is arterial in its color, nor does it lose its bright hue until the nutritive actions and the waste of the body are restored, and effete matters are evolved in the chemical laboratory of nature.

It is admitted that the danger of bilious affections is just in proportion to the continuance or duration of this ardent fever. Happily for us, in our autumnal diseases, the violence of the fever soon gives way, the molecular movements are restored to great activity, effete matters are abundantly separated, the emaciation is rapid and the recovery equally so. In yellow fever—a disease of one paroxysm—a phenomenon distinctive in itself—unhappily the result is too often different. In this affection the curative powers of nature seem fettered; nor can she, in too many instances, break the bonds by which her powers are restrained. In all grave cases we witness from the commencement a gradual decrease, oftentimes a speedy cessation of secretions of all the organs and tissues of the whole system. While there exist in excess, most probably, all the elements required for elimination and secretion, yet in a simple but fatal case of the disease, we find the energies of vital chemistry—the action and reaction between the solids and fluids—greatly impaired, soon destined to be suspended—sometimes from the beginning abolished, never again to be resumed. In this disease, most generally, the fever is very intense, the whole body is in a state of great irritation, the cause, or the malady, or both united, arresting molecular actions; the carbon of the body and its compounds become imprisoned, as it were—and hence in the withdrawal of the antidote in the human system of oxygen, whilst the last irritates and goads on the heat, we have presented to us a most violent, but in the view we take of it, a simple and intelligible form of disease.

We occasionally, but very rarely in this region of country, witness—generally in early summer—an affection which, in all its symptoms clearly resembles yellow fever. In its onset, progress, ardency of the fever—in the racking pains of head, back and limbs—in the light, arterial color of the blood when drawn from the veins—in the bloated tissues, anxious, bright eye, gradual suspension of secretions, ending in a virtual exsiccation of the whole system—we have the counterpart of the disease called yellow fever, and here we have the whole body in a condition analogous to that which prevails in an inflamed surface, only in the one case there is diffused irritation, in the other a local inflammation—no carbon existing in either case to neutralize the superabundant oxygen of the blood. If such statement be true, it appears to me to furnish the means for explaining the phenomena of yellow fever, as well as of that peculiar form of bilious fever, simulating the yellow, above alluded to.

The evidences of the imperfection of medical science surround us on all sides, but the discrepancy of opinions of the ablest in the profession on the most important points—and especially at the bedside of the patient—should inspire the honest follower of the healing art with profound mortification. Is it true that disease is ever to be a subject of disputation—one of the arcana of nature—and that the physician has but to supply himself with patent medicines to await the maladies we may encounter? Such disgrace seems to threaten the medical profession. Every day some new remedy is highly recommended. In a few months or years it is laid aside or substituted by another whose fate is the same. In our ignorance we are striving, with all praise, to make our necessarily false practice, and imperfect observation the means for the discovery of truth, and we are driven to confess that truth has mocked us in our researches, and to admit that our science has made no advance. We realize the fact that our art is but a collection of imperfect, approximative truths. And in the absence of positive knowledge of the constituent elements of the body, and of the chemical changes which disease engenders, we are most likely destined to grope our way in darkness, mere *empirics*. But there are certain phenomena which may be appreciated by the mind as facts or truths.

It may be stated that all fevers necessarily result from the introduction into the body of something deleterious—that the deleterious or morbid matter does not produce disease so long as the preservative forces expel it from the system. But on continued exposure to the cause, (and illustratiue examples are seen in every epidemic—contagious or infectious.) Nature finally subcumbs—the poisonous influences affect the whole body—and fever results. In the typhoid we see immediately instituted a rapid waste of the solids—for weeks and weeks an excessive process of emaciation goes on. Without food or restorative elements from without, the blood is yet preserved in a condition to sustain life, until finally the solids are liberated from the poison and its effects, and convalescence results. So in bilious fever: after a few hours of intensity the fever abates—the suspended waste of the body is resumed, and we have a speedy recovery, unless engorgements occur, dependent on the superábundance of eliminated matters, and the weakened state of the engorged organs. In a simple case of yellow fever, very often the nutritive, in eliminating the excreting powers of the body gradually become suspended, soon no waste of the solids takes place, the blood becomes surcharged with oxygen, and we witness the most extraordinary ensemble of symptoms, until death closes the scene.

If the above statements bear the aspect of truth, they merit the attention of all students and medical practitioners.

